# *In Vivo* Targeting Replication Protein A for Cancer Therapy

**DOI:** 10.3389/fonc.2022.826655

**Published:** 2022-02-18

**Authors:** Pamela S. VanderVere-Carozza, Navnath S. Gavande, Shadia I. Jalal, Karen E. Pollok, Elmira Ekinci, Joshua Heyza, Steve M. Patrick, Andi Masters, John J. Turchi, Katherine S. Pawelczak

**Affiliations:** ^1^ Department of Medicine, Indiana University School of Medicine, Indianapolis, IN, United States; ^2^ Department of Pharmaceutical Sciences, Wayne State University College of Pharmacy and Health Sciences, Detroit, MI, United States; ^3^ Herman B. Wells Center for Pediatric Research, Departments of Pediatrics, Pharmacology and Toxicology, Medical and Molecular Genetics Indiana University Simon Comprehensive Cancer Center, Indianapolis, IN, United States; ^4^ Department of Oncology, Wayne State University School of Medicine and Barbara Ann Karmanos Cancer Institute, Detroit, MI, United States; ^5^ Indiana University Cancer Center, Indiana University School of Medicine, Indianapolis, IN, United States; ^6^ NERx BioSciences, Indianapolis, IN, United States

**Keywords:** DNA repair inhibitors, Replication Stress Response, Replication Protein A, DNA damage response, DNA repair and cancer

## Abstract

Replication protein A (RPA) plays essential roles in DNA replication, repair, recombination, and the DNA damage response (DDR). Retrospective analysis of lung cancer patient data demonstrates high RPA expression as a negative prognostic biomarker for overall survival in smoking-related lung cancers. Similarly, relative expression of RPA is a predictive marker for response to chemotherapy. These observations are consistent with the increase in RPA expression serving as an adaptive mechanism that allows tolerance of the genotoxic stress resulting from carcinogen exposure. We have developed second-generation RPA inhibitors (RPAis) that block the RPA–DNA interaction and optimized formulation for *in vivo* analyses. Data demonstrate that unlike first-generation RPAis, second-generation molecules show increased cellular permeability and induce cell death *via* apoptosis. Second-generation RPAis elicit single-agent *in vitro* anticancer activity across a broad spectrum of cancers, and the cellular response suggests existence of a threshold before chemical RPA exhaustion induces cell death. Chemical RPA inhibition potentiates the anticancer activity of a series of DDR inhibitors and traditional DNA-damaging cancer therapeutics. Consistent with chemical RPA exhaustion, we demonstrate that the effects of RPAi on replication fork dynamics are similar to other known DDR inhibitors. An optimized formulation of RPAi NERx **329** was developed that resulted in single-agent anticancer activity in two non-small cell lung cancer models. These data demonstrate a unique mechanism of action of RPAis eliciting a state of chemical RPA exhaustion and suggest they will provide an effective therapeutic option for difficult-to-treat lung cancers.

## Introduction

The DNA damage response (DDR) is composed of a complex network of DNA repair and cell signaling pathways that are critical toward maintaining genomic stability. Dysfunctional DDR causes damage to the genome that results in genomic instability, providing a selective advantage over normal cells and enabling rampant proliferation and survival. This genomic instability frequently arises from mutations of certain cell cycle and DDR genes, which in turn creates an increased dependency on other components of the DDR network. This reliance on specific DDR machinery can make cancer cells more vulnerable to therapies targeting DDR components. Certain drugs, like the popular poly (ADP-ribose) polymerase (PARP) inhibitors, can take advantage of targeting cancers with specific known DDR mutations and can impart therapeutic benefit through a synthetic lethality approach (1). Recent evidence also suggests that the DDR is involved in activation of the innate immune response, suggesting that DDR inhibitors combined with immunotherapy may have anticancer activity (2). Other agents targeting specific DDR signaling molecules have shown single-agent and combination activity (3).

Oncogenic replication stress (RS) coupled with DDR blockade results in local effects at the replication fork and global effects on cell cycle and signaling that ultimately result in replication catastrophe (RC) and cell death. The human single-stranded DNA (ssDNA) binding protein, replication protein A (RPA), is a critical regulator of the DDR, with depletion of active RPA or “RPA exhaustion” driving RC and cell death. RPA is the major eukaryotic ssDNA binding protein, and its level and activity are tightly regulated. High levels of ssDNA resulting from DDR inhibition can exhaust cellular RPA such that there is insufficient RPA–DNA binding capacity to engage all the ssDNA generated. The lack of RPA available to protect ssDNA then renders DNA susceptible to digestion by nucleases resulting in DNA strand breaks at replication forks, RC, and cell death (4, 5). We have targeted this crucial DNA metabolic pathway required for genome stability and maintenance *via* small molecule inhibitors (SMIs) that block the RPA–DNA interaction.

First-generation RPA inhibitors (RPAis) were developed and have been extensively characterized with respect to potency and mechanism of action (6–8). Predecessor RPAi TDRL-551 **(551)** displays *in vivo* activity in lung cancer xenograft models. In an effort to determine if lowering the RPA threshold with **551** would result in a synergy with DNA-damaging agents like platinum (Pt)-based drugs, *in vivo* efficacy studies were performed in non-small cell lung cancer (NSCLC) xenograft models. Combinatorial experiments with Pt were conducted with a reduction of both carboplatin and **551** doses to ensure a window to observe potential synergy. Single-agent activity was observed, as well as a greater than additive effect on tumor growth delay with the carboplatin–**551** combination compared to each agent alone. In addition, **551** displays *in vitro*, cellular, and *in vivo* anticancer activity and synergy with cisplatin. Despite the effectiveness of **551** in preclinical studies, certain chemical moieties of the molecule represented chemical liabilities for clinical readiness of the drug. A series of second-generation inhibitors was generated and optimized for solubility, stability, and cellular uptake (9). A morpholino derivative, NERx **329** (**329**), demonstrated enhanced solubility and cellular uptake with superior physicochemical properties. The chemical modifications resulting in ideal drug-like characteristics in the **329** molecule are expected to vastly improve cellular potency, the *in vivo* anticancer activity, and general clinical readiness of the drug. Here, we report the cellular effects and *in vivo* studies completed with **329** and introduce a novel formulation strategy that dramatically improves bioavailability of **329**.

## Materials and Methods

### Replication Protein A Inhibitors

RPAis 329 and 2004 were synthesized and characterized as previously described (9).

### Electrophoretic Mobility Shift Assay (EMSA)

EMSAs were performed as previously described (9). Briefly, reactions were conducted in 20 mM HEPES (pH 7.8), 1 mM DTT, 0.001% NP-40, and 50 mM NaCl. RPAis were suspended in 100% dimethlysulfoxide (DMSO), and DMSO concentration in the final reaction mixture was constant at less than 5%. Purified full-length RPA (120 ng) was incubated with the indicated RPAi or vehicle in reaction buffer for 30 min before the addition of the [^32^]P-labeled 34-base ssDNA probe. Reactions were incubated for 5 min at room temperature, and products were separated *via* 6% native polyacrylamide gel electrophoresis. The bound and unbound fractions were quantified by phosphor-imager analysis using ImageQuant software (Molecular Dynamics, CA), and data were fit by non-linear regression using GraphPad Prism.

### CCK-8 Viability Assays

Cell lines were obtained from ATCC and maintained as monolayer cultures in RPMI 1640 medium (H460) or Dulbecco’s Modified Eagle's Medium (DMEM) (A549) supplemented with 10% fetal bovine serum. H460 and A549 cells were plated at 2.5 × 10^3^ cells/well and A2780 and GCT27 cells plated at 5 × 10^3^ cells/well in a 96-well plate and incubated for 18–24 h prior to treatments. Cells were treated with the indicated concentration of RPAi for 48 h. The vehicle (DMSO) concentration was held constant at 1%. Cell metabolism/viability was assessed by a mitochondrial metabolism assay (CCK-8) as we have described previously (10). The generation of the water-soluble formazan product by cellular dehydrogenases is proportional to the number of living cells. Following incubation with CCK-8 reagent, absorbance was measured at 450 nm with a BioTek Synergy H1 plate reader. Values were compared to those of vehicle-treated controls to determine percent viability, and the results represent the average and SEM of triplicate determinations.

### Apoptosis Assay

Apoptosis induction was determined by activation of Caspases 3 and 7 using the CellEvent™ Caspase-3/7 Green Detection Reagent (Invitrogen). H460 cells were plated at 5 × 10^3^ cells/well in black 96-well plates with clear bottoms (Costar) and incubated for 24 h prior to treatments. Cells were treated with the indicated concentration of RPAi or cisplatin for 24 h. The vehicle (DMSO) concentration was held constant at 1% for RPAi treatments. For caspase 3/7 detection, medium was removed and replaced with phosphate buffered saline (PBS) containing 5% fetal bovine serum (FBS) and 2 µM CellEvent™ Caspase-3/7 Green Detection Reagent. Cells were incubated at 37°C/5% CO_2_ for 1 h, and fluorescence intensity was measured in a BioTek Synergy H1 plate reader (excitation/emission 485/528). Images were captured with an Evos FL2 Auto microscope (Invitrogen) using a 10× objective.

### Cell Viability in 60 Cancer Cell Lines

In this study, 90-µl cell suspensions were seeded in 96-well plates in respective culture medium with a final cell density of 4 × 10^3^ cells/well and incubated overnight. Here, 10× solution of **329** (top working concentration: 40 µM of test article in media with 3.16-fold serial dilutions to achieve 9 dose levels) was prepared, and 10 µl of drug solution or culture medium containing 0.5% DMSO (vehicle control) was added to the plate (triplicate for each drug concentration). Plates were incubated for 72 h at 37°C with 5% CO_2_ and then measured by CellTiter-Glo assay (Promega). Briefly, plates were equilibrated at room temperature for 30 min, and 50 µl of CellTiter-Glo reagent was added to each well. Contents were mixed for 5 min on an orbital shaker to induce cell lysis, and plates were further incubated at room temperature for 20 min to stabilize the luminescent signal. Luminescence was recorded using EnVision Multi Plate Reader. Percent cell growth was calculated relative to DMSO-treated cells (vehicle control), and the data were fit using non-linear regression analysis (GraphPad PRISM) to calculate cellular IC_50_.

### DNA Fiber Analysis

Analysis of DNA replication intermediates was performed as previously described with minor modifications (11, 12). H460 cells were seeded in 6-well plates at a density of 2 × 10^5^ cells. The following day, cells were labeled with iodo-deoxyuridine (IdU) (20 µM) for 20 min, followed by treatment with hydroxyurea (HU) (2.5 mM) for 60 min, then released into chloro-deoxyuridine (CldU) (200 µM) for 20 min, followed by treatment with ATR inhibitor (ATRi) VE-822 (2 µM, Selleckchem, S8807) for 2 h or RPAi **329** (50 µM) for 2 h. After harvesting, the cells were resuspended in PBS at a concentration of 1,000,000 cells/ml, and 2 µl of the cell suspension was mixed with 8 µl of lysis buffer (200 mM Tris-HCl pH 7.5, 50 mM EDTA, 0.5% SDS) on a Superfrost Plus microscope slide (Fisher Scientific). After 6 min of incubation, the slides were tilted at a 45-degree angle to allow cell lysates to slowly run down the slide. After air-drying, the slides were fixed in methanol:acetic acid (3:1) and stored at 4°C. DNA fibers were denatured with 2.5N HCl for 1 h, washed with PBS, and blocked with 5% BSA in PBS-T (PBS + 0.1% Tween-20) for 1 h. DNA fibers were incubated with rat anti-BrdU antibody (1:50, Abcam, ab6326) for CldU and mouse anti-BrdU antibody (1:50, BD Biosciences, 347580) for IdU in a humid chamber at 37°C for 1 h. After washing, slides were incubated with secondary antibodies anti-rat Alexa 488 (1:100) and anti-mouse Alexa 568 (1:100) at room temperature for 45 min. Excess antibodies were removed by washing with PBS-T 3 times. After air-drying, the slides were mounted onto a coverslip with mounting medium. Fiber tracts were imaged with a Nikon epifluorescence microscope using a 40× oil immersion objective, and 100 fibers for each group were analyzed in ImageJ where the ratios of CldU : IdU were compared using pixel length. Data were analyzed by ANOVA with Bonferroni test for multiple comparisons.

### Combination Studies

To assess synergy, the combination index (CI) was determined as described by Chou-Talalay as we have previously described (8). Briefly, H460 cells were treated with RPAi and the indicated agent alone and in combination. The range of treatment was dependent on the IC_50_ of each agent, and the range was ¼ to 3 × IC_50_. The data from both the single-agent treatments and the combination treatment were used to calculate the CI and plot this value as a function of the fraction of cells affected (Fa). A CI of >1 indicates antagonism between the two agents, while a CI <1 indicates synergy. A CI of 1 demonstrates an additive effect.

### Pharmacokinetics

A method to quantify **329** from plasma has been developed using an internal standard, liquid–liquid extraction, and HPLC-MS/MS. Mouse plasma samples were prepared from treated mice at the indicated times frozen at -80°C until analysis. Plasma samples were thawed (20 µl) and transferred to polypropylene tubes, and the internal standard is added (20 µl of 0.1 ng/µl). Samples were diluted in 0.1 M phosphate buffer (pH = 7.4) and equal volume of methyl tertiary butyl ether. The samples were mixed and centrifuged at 12,000×g for 5 min, and the supernatant was transferred to a clean polypropylene tube. The solvent was evaporated to dryness, brought up in mobile phase analyzed by HPLC-MS/MS (ABSciex 4000). The mobile phase is delivered *via* gradient using acetonitrile and 0.1% formic acid on an Agilent Zorbax 300SB-C8 150 × 4.6 mm, 5-µm column. The mass spectrometer utilized an electrospray ionization probe run in positive mode. Multiple reaction monitoring was employed with Q1/Q3 (m/z) transitions for **329** at 718.2/128.1 and 687.3/128.1 for the internal standard. The lower limit of quantification is 0.1 ng/ml using 20 µl of plasma.

### *In Vivo* Analyses

To assess anticancer efficacy, the hind flanks of 60 8–10-week-old Nod SCID gamma (NSG) mice were implanted with the indicated cells (~2 × 10^6^) in Matrigel. Tumor volumes were monitored by electronic caliper measurement [tumor volumes = length × (perpendicular width)^2^ × 0.5]. NSG studies were approved by the Institutional Animal Care and Use Committee at Indiana University School of Medicine. Male NSG (NOD‐scid/IL2Rg^null^) mice (*In Vivo* Therapeutics Core Facility, IU Simon Comprehensive Cancer Center, Indianapolis, IN, USA) were used and housed in a pathogen‐free facility at IUSM LARC. Mice with tumors of approximately 100 mm^3^ were randomized into individual treatment arms. The indicated RPAi was formulated and administered *via* intraperitoneal injection (IP) at the indicated times. Tumor volumes were monitored biweekly as indicated, and the results are presented as the average tumor volume ± standard error of the mean for each group. The number (n) for each experiment is presented in the figure legend.

## Results

### Retrospective Analysis of Replication Protein A Expression in Lung Cancer

**Figure 1:**
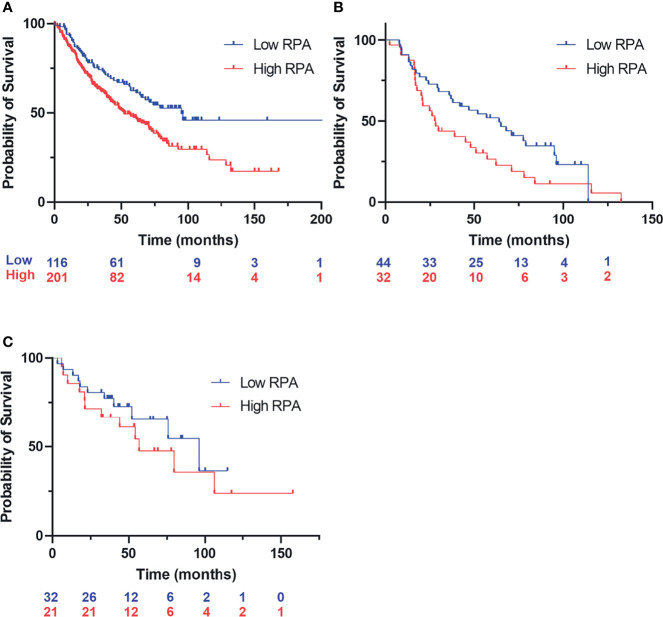
Kaplan–Meyer retrospective analysis of overall survival as a function of replication protein A (RPA) gene expression in non-small cell lung cancer (NSCLC). Blue numbering indicates patients with low RPA expression, red numbering indicates patients with high RPA expression. Analysis represents a 500-patient cohort from the caARRAY, with optimized cutoff. **(A)** Former and current smokers. HR = 1.63 (1.17–2.28) log-rank: p = 0.0035. **(B)** Former and current smokers who received chemotherapy. HR = 1.69 (1–2.86) log-rank: p = 0.049. **(C)** Never smokers.

Considering the model of RPA exhaustion limiting the DDR to exogenous damage and replication stress, we sought to determine how the expression of RPA impacted survival in lung cancer. We selected lung cancer, as lung epithelial cells are continuously exposed to a wide array of potentially carcinogenic agents, a situation exacerbated by smoking and second-hand smoke exposure. To assess the potential clinical utility of RPA inhibition, we performed a retrospective analysis of gene expression data in lung cancer as a function of smoking history and response to chemotherapy treatment. In current and former smokers, the data reveal that high RPA expression is a negative prognostic biomarker correlating with worse overall survival (**Figure 1A**). This difference in survival as a function of RPA expression was also observed when selecting patients who received adjuvant chemotherapy that often includes Pt-based DNA-damaging agents (**Figure 1B**). These data demonstrate that low RPA expression is predictive of a better therapeutic response. In the analysis of never smokers (**Figure 1C**), no correlation between RPA expression and survival was observed. Importantly, this patient population is a collection of heterogeneous cancer phenotypes that is characterized by a higher level of driver mutations in growth signaling pathways, and as such, these never smokers are expected to have received targeted kinase inhibitor therapy. The finding that RPA expression level does not impact survival is therefore not surprising. Collectively, these data suggest that potential genotoxic damage induced by smoke exposure induces reliance on RPA expression to protect against genotoxic stress that, if reversed, could impact survival.

### Chemical Inhibition of Replication Protein A and Mechanisms of Cell Death

**Figure 2:**
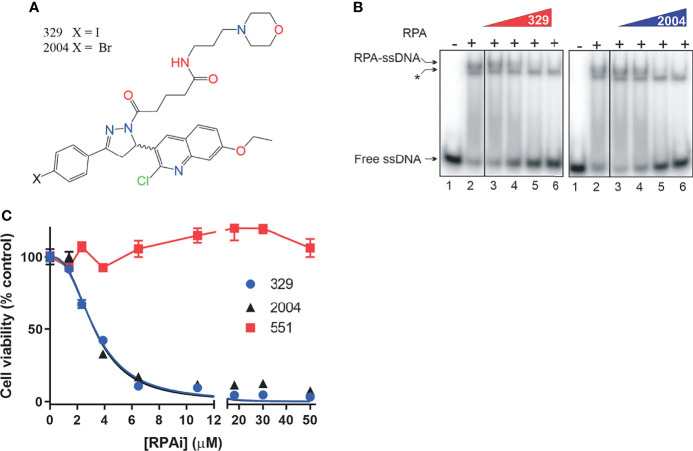
Replication protein A inhibitor (RPAi) inhibitory activity. **(A)** Chemical structure of RPAi’s **329** and **2004**. **(B)** EMSA analysis of RPA–DNA interaction inhibition by **329** and **2004**. Lanes 3–6 in each panel contain 6.25, 12.5, 25, and 50 µM of the indicated RPAi, respectively. The * indicates the position of the *Escherichia coli* SSB–single-stranded DNA (ssDNA) complex that serves as an internal specificity control. **(C)** Cell viability of H460 NSCLC cells in response to **329** and **2004**.

Our previous analyses of reversible RPAis revealed both *in vitro* and *in vivo* activity, but chemical liabilities limited their broad utility in cell-based and *in vivo* assays (6, 8). We have further optimized the **551** candidate to generate candidate RPAi **329** and a derivative **2004** (**Figure 2A**) that possess potent RPA inhibitory activity *in vitro*, *in vivo*, and in cellular assays (**Figures 2B, C** **)**. The data also show that the compounds are specific for inhibiting the RPA ssDNA interaction, as the interaction of *Escherichia coli* single strand binding protein (SSB) with ssDNA as indicated is not impacted by the RPAis. These compounds also display excellent solubility, cellular uptake, and physicochemical properties (9). As the addition of a propyl-morpholino to the oxopentoic acid moiety increased solubility and cellular uptake, we sought to assess single-agent cellular anticancer activity in the H460 NSCLC cell line (**Figure 2C**). The data demonstrate that **329** and **2004** possess potent single-agent activity compared to the **551** predecessor as assessed by CCK-8 metabolic assay.

**Figure 3:**
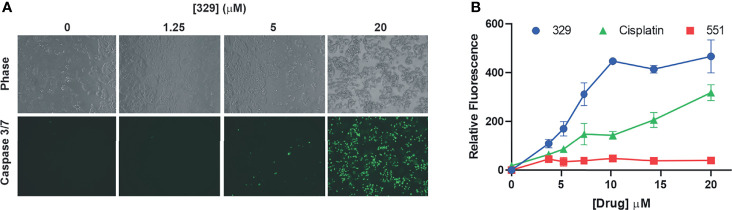
The **329** induction of apoptotic cell death. **(A)** Analysis of caspase 3/7 activity in H460 cells following 24 h of treatment with 1% DMSO or the indicated concentrations of **329**. Fluorescence images were captured as described in the *Materials and Methods*. **(B)** Quantification of caspase 3/7 activity. Fluorescence was measured in 96-well plates using a Biotek Synergy H1 plate reader following 24-h incubation with the indicated drugs and concentrations.

Predecessor reversible RPAis **505** and **551** also displayed single-agent anticancer activity, although this was not accompanied by caspase activation or annexin V/PI positivity, suggesting a non-apoptotic mechanism of cell death (6). The increased cellular uptake and potency displayed by the morpholino-containing compound **329** prompted us to revisit this activity. Using the activation of caspases 3 and 7 as a readout, we demonstrate that **329** induces cell death *via* a classical apoptotic pathway (**Figure 3**), and the activation of caspases 3 and 7 clearly distinguishes it from predecessor compound **551**. Importantly, **551** does show decreased viability in clonogenic survival assays at the concentrations tested. The inability to detect caspase activity suggests that this is a distinguishing characteristic between the two (8). The titration analyses assessing apoptosis correlated with the corresponding CCK-8 viability curves and show the presence of a modest threshold. Assessment of apoptosis at 48 h was similar to 24 h in terms of the titration, though the maximum signal detected was higher, as expected.

**Figure 4:**
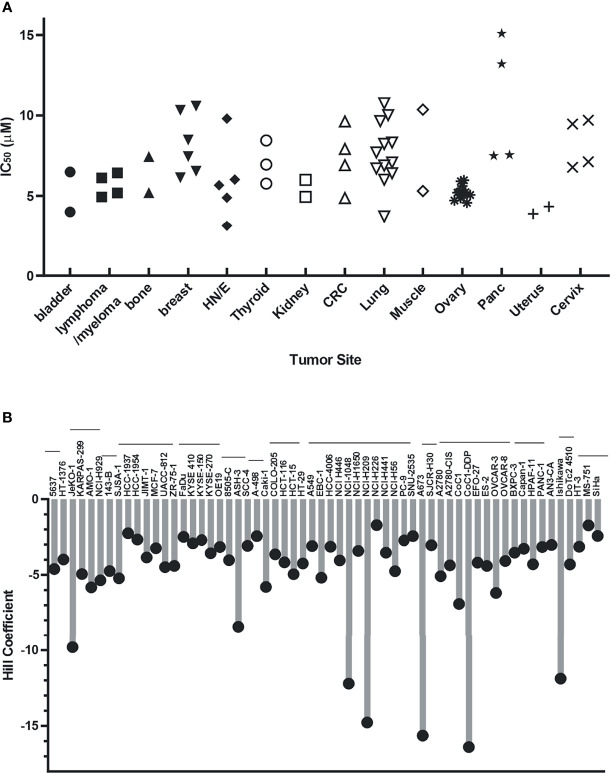
Cellular activity of **329** in 60 cancer cell lines. Cell lines were treated with a 4-log range of replication protein A inhibitor (RPAi) **329** for 72 h. Cell viability was assessed using CellTiter-Glo luminescent viability assay. The data represent the average of triplicate treatments, and the data were fit using non-linear regression analysis to calculate cellular IC_50_s. **(A)** IC_50_ results from each cell line grouped by tumor type. **(B)** Hill coefficients for individual cell lines. The horizontal lines above cell line names indicated the tumor sites in the order depicted in panel **(A)**.

Analyses of single-agent activity of compound **329** in 60 discrete cancer cell lines across a variety of solid tumors revealed similar findings. A range of IC_50_ values were obtained, with most falling between 5 and 10 μM and largely independent of tumor site (**Figure 4A**). Certain uterine, lung, and esophageal cancer cell lines were the most sensitive, while pancreatic adenocarcinomas tended to be more resistant. Interestingly, the Hill coefficients spanned a much larger range (**Figure 4B**), which did not necessarily correlate with the potency as measured by IC_50_. Certain lung, muscle, ovarian, and cervical cancer lines were characterized by the lowest Hill coefficients. These data are consistent with the tumor agnostic nature of RPA inhibition and a mechanism of action involving a threshold of measurable cytotoxic sensitivity.

**Figure 5:**
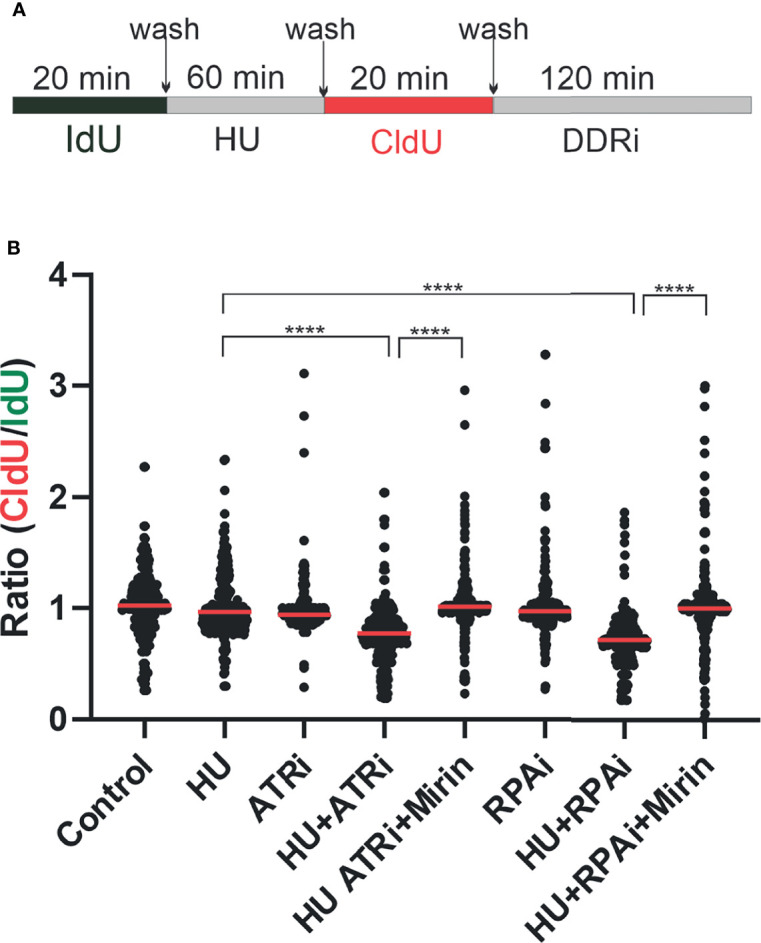
Replication protein A inhibitor (RPAi) impact on replication fork dynamics. **(A)** Schematic depiction of experimental design. DNA was pulse-labeled with IdU for 20 min. After IdU removal, replication forks were stalled by the addition of HU or left to replicate with vehicle treatment. HU was removed, and replication was labeled with CldU. Following CldU, cells were treated with the DDRi or vehicle. **(B)** Quantification of results from DNA fiber analysis in H460 cells treated with the indicated agents. HU was used at a final concentration of 2.5 mM, the ATRi VE-822 at 2 µM, and the RPAi **329** at 50 µM. Data presented are combined from three individual experiments (100 fibers analyzed per experiment; 300 fibers total). Red bar indicates the median value of CldU/IdU. Data were analyzed by ANOVA with Bonferroni test for multiple comparisons (****p < 0.0001).

A further measure of altered DDR induced by RPA inhibition is the degradation of replication forks upon stalling and RPA exhaustion. We therefore assessed replication fork dynamics and nascent strand degradation using DNA fiber analysis. The treatment scheme is depicted in **Figure 5A**. We first pulse-labeled replicating DNA with IdU for 20 min. After IdU removal, replication forks were stalled by the addition of HU or left to replicate with vehicle treatment. HU was removed and replication labeled with CldU. Then, CldU cells were treated with the DDRi or vehicle. The data obtained are presented in **Figure 5B**. As expected, minimal effects were observed with ATRi or RPAi alone. However, in cells that received HU and then either ATRi or RPAi, a significant decrease in CldU/IdU signal was observed. These data suggest that the addition of DDRi after fork stalling by HU results in nascent strand degradation at stalled replication forks. Importantly, this decrease observed was reversible by mirin, an inhibitor that blocks degradation of the forks. Importantly, the effect of RPAi was similar to ATRi, as expected for targets in the same pathway. These data suggest that DDR checkpoint abrogation by ATRi or RPAi and a subsequent increase in the presence of unprotected ssDNA in S-phase result in replication fork instability and nascent strand degradation.

### Therapeutic Combinations

**Figure 6:**
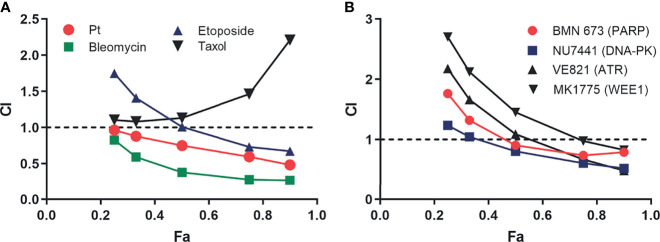
Analysis of replication protein A inhibitor (RPAi) **329** combination treatment. **(A)** Chou-Talalay analysis of combination with chemotherapeutics. The combination index **(CI)** is plotted as a function of the fraction of cell affected (Fa) for each treatment combination of the **329. (B)** Chou-Talalay analysis of combination with DDR-targeted agents as described in panel **(A)**.

Considering RPA’s role in numerous DNA metabolic processes, we determined how inhibition of RPA impacts sensitivity to a variety of DNA-damaging chemotherapeutics that induce different types of damage. Interestingly, we observe synergy, as indicated by a CI <1 at 0.5 or higher fraction of cells affected, with agents that cause replication stress, bulky lesions, and DNA double-strand breaks (**Figure 6A**), whereas no synergy was observed with paclitaxel, a non-DNA-damaging therapeutic. These results suggest that the cytotoxic effects of RPAis may be mediated by a broader effect on the DDR as opposed to suppression of individual replication and repair pathways. Considering these data, we suspected that RPA inhibition would synergize with other DDR-targeted therapeutics to block multiple pathways within the more broadly concerted DDR. We therefore assessed synergy of RPAis with a series of DDR-targeted agents that are currently in clinical trials (**Figure 6B**). The data demonstrate that modest synergy is observed with each agent in the H460 NSCLC cell model, with exception of the Wee1 inhibitor. Interestingly, we did observe modest synergy with the PARPi BMN637 in BRCA wild-type cells despite the relatively limited activity seen with single-agent PARPi in these cells. Not surprisingly, we have demonstrated a greater degree of synergy between RPAi and PARPi in BRCA1 null cells compared to BRCA wild-type cells (13). Interestingly, both ATR and DNA- dependent protein kinase (PK) inhibition were more effective when used in combination with RPAi treatment, suggesting that either inhibition of parallel pathways or sequential inhibition of a single pathway in the case of ATR contributes to enhanced increased anticancer activity. Wee1 inhibition however was antagonistic or additive with RPAi over the entire range of cells affected that places its activity downstream of RPA as expected.

### *In Vivo* Analyses

Toward the goal to identify efficacious and safe RPAi **329** treatment regimens, we conducted single-agent screening in two lung cancer cell line-derived xenograft models. Predecessors to **329** and **2004**, compounds **505** and **551**, possessed modest *in vivo* activity (8). Having optimized cellular uptake and solubility *via* the addition of the propyl morpholino in **329** and **2004**, we sought to determine how these modifications impact *in vivo* anticancer activity using two NSCLC models, A549 adenocarcinoma and H460 large cell carcinoma. Analysis of toxicity revealed that safe dosing could be achieved up to 200 mg/kg with no overt toxicity when formulated as a suspension in DMSO/Tween and no significant loss in body weight similar to predecessor compounds. Assessment of kidney function also showed no differences from vehicle controls (data not shown). Interestingly, we observed only modest single-agent anticancer activity in both models with differing dosing regimens of **329** and **2004** (14). That the modest *in vivo* activity is in fact similar to that observed for the **551** predecessor compound (8) was surprising in light of the increases in RPA inhibitory activity *in vitro*, increase in cellular uptakes (9), and dramatically increased activity in tissue culture models. This result suggested that the morpholino addition to **551** to generate **329** could be negatively impacting bioavailability. Analyses of intrinsic clearance and half-life were conducted in mouse and rat microsomes (**Table 1**). Here, **329** displayed favorable rates of clearance in mouse microsomes, ~43 μl/min/mg. These values are less than the rate of 48, which is considered rapid clearance. In rat microsomes, rates of ~64 were obtained for 329 and are less than the rapid rate of 71. Half-lives of 20–40 min in mice and rats are also well within range for these *in vitro* clearance studies, suggesting that **329** was not limited by these parameters. Comparative analyses of PK parameters with **329** *vs*. **551** in the DMSO/Tween formulation revealed a significant reduction in AUC and C_max_ with **329** compared to **551** (data not shown).

**Table 1:** Plasma stability and clearance.

		329
	Mouse	
t_1/2_	(min)	43.9
CLint	(uL/min/mg)	31.7
	Rat	
t_1/2_	(min)	21.7
CLint	(uL/min/mg)	63.8

Analyses of intrinsic clearance and half-life were conducted in mouse and rat microsomes.

### Formulation of NERx 329

**Figure 7:**
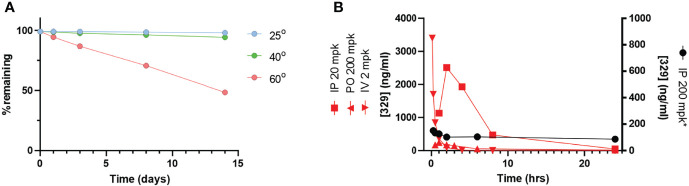
The **329** *in vivo* analysis. **(A)** Stability analysis. Compound stability was assessed over a 14-day time period at varying temperatures as indicated. **(B)** Pharmacokinetic analysis. Time course of drug plasma concentration over 24 h following drug administration as indicated in legend.

With favorable potency, cellular activity, plasma stability, and a clear deficit in PK, we initiated a series of studies to assess and optimize a formulation of **329** for *in vivo* bioavailability. Here, **329** solubility was assessed in a series of additives, excipients, and co-solvents to identify initial favorable vehicles (**Table 2**). The surprising result was that **329** was highly soluble in N-methylpyrilidone and displayed moderate solubility in oleic acid, propylene glycol, and PEG400. A series of different formulations were assessed. The final formulation consisted of polysorbate 80, N-methyl-2-pyrrolidone (NMP), propylene glycol, and PEG 400 (+1.1 mol. eq. HCl added as 12 M HCl), and **329** was soluble up to 20 mg/ml, and 10-fold dilution in PBS was well dispersed with minimal precipitation. Fourteen-day stability assessments were conducted with this formulation, and the data demonstrate that **329** is very stable up to 40°C, while degradation was observed with extended incubations at 60°C (**Figure 7A**) Calculation of the T90, time to reduce active agent to 90%, was calculated for each temperature and extrapolated to 5°C where **329** is predicted to be stable for over 5 years and at room temperature for over 3 months (**Table 3**). Based on these data, the lead formulation is deemed stable for preparation and storage of **329** at room temperature to support *in vivo* studies.

**Table 2:** Vehicle/excipient screen.

Vehicle	Solubility (mg/m I)
Water	<1
50 mM sodium acetate pH 4	<1
30% SBECD in water	<1
Oleic acid	2.4
Cremophor EL	<1
Labrasol	1.1
Propylene glycol	1.3
Polyethylene glycol 400	1.2
NMP	70.4
Polysorbate 8O	<1
Ethanol	<1

A series of additives, excipients, and co-solvents were assessed in basic formulation studies to optimize bioavailability.

**Table 3:** Stability analysis.

Condition	t90* (days)
60°C	3.2
40°C	26
25°C	140
5°C (extrapolated)	2100

The chemical stability of 10 mg/ml of NERx **329** in lead formulation was assessed at 25°C, 40°C, and 60°C for a period of 2 weeks.

### Pharmacokinetic Analysis of 329 in Optimized Formulation

PK parameters were assessed in a series of studies in immunocompetent mice both IV and IP (**Figure 7B** and **Table 4**). Data demonstrate that IP delivery with the new formulation at 200 mg/kg is enhanced, and results showed significantly improved values including C_max_, T_max_, and AUC. The half-life was also well within acceptable range (9 h). However, dosing at 200 mg/kg in the new formulation resulted in increased toxicity. Considering the increased exposure as a result of drastically improved PK parameters, increased toxicity is not surprising. Interestingly, reducing the dose to 20 mg/kg results in a similar C_max_ but reduced AUC as a result of T_1/2_ and T_max_ differences. IV dosing was performed to allow calculation of absolute bioavailability of 0.84 at 20 mg/kg.

**Table 4:** Pharmacokinetic analysis of **329**.

Parameter	Unit		Value		
Route/Vehicle/dose	mg/kg	|lP/DMSO/200	IP/NMP/200	IP/NMP/20	IV/NMP/2
Lambda_z	1/h	0.02	0.07	0.11	0.21
t1/2	h	30.86	9.311	6.30	3.28
Tmax	h	0.25	8.00	2.00	0.50
Cmax	ng/ml	92.11	2913.57	2511.65	840.89
Clast_obs/Cmax		0.305	0.315	0.002	0.003
AUC 0-t	ng/ml*h	967.99	52010.59	16425.20	1396.47
AUC 0-inf_obs	ng/ml*h	2218.00	64333.98	16474.36	1407.62
AUC 0-t/0-inf_obs		0.44	0.81	1.00	0.99
AUMC 0-inf_obs	ng/ml*h^2	95023.52	947545.56	98489.24	1897.83
MRT 0-inf_obs	h	42.84	14.73	5.981	1.35
Vz/F_obs	(mg/kg)/(ng/ml)	4.015	0.172	0.011	0.007
CI/F_obs	(mg/kg)/(ng/ml)/h	0.0902	0.0128	0.0012	0.0014

Drug formulated in the optimal NMP solution was assessed for IV or IP delivery at varying doses, as indicated.

### *In Vivo* Analysis of 329 in Optimized Formulation in Non-Small Cell Lung Cancer (NSCLC) Xenograft Models

**Figure 8:**
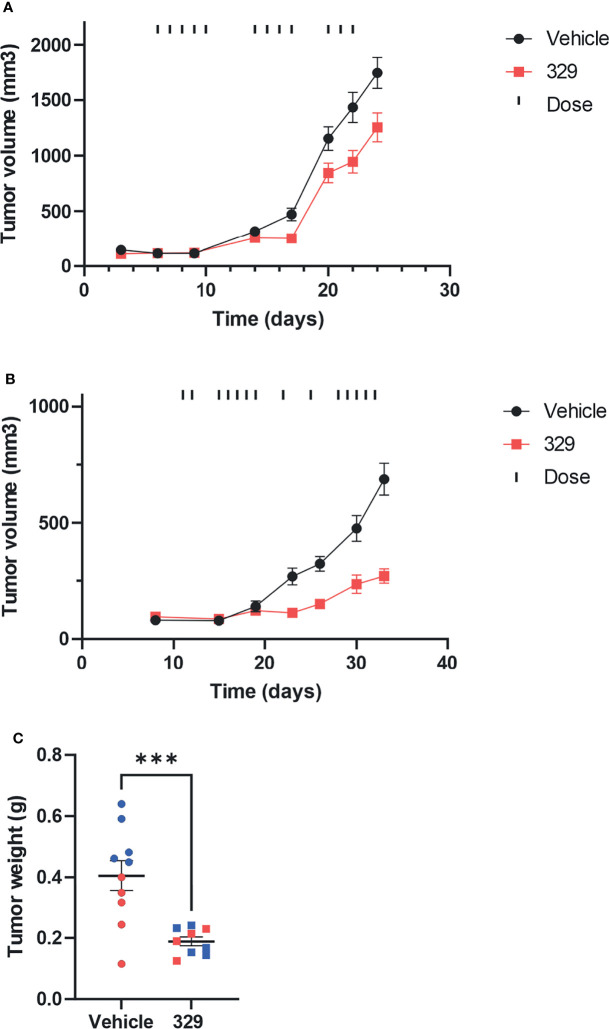
*In vivo* analysis of anticancer activity of **329**. **(A)** Anticancer activity was assessed in human H460 NCSLC tumor xenografts in NOD/SCID mice. Mice were implanted subcutaneously on day 1 with H460 NSCLC cells, tumors were measured by calipers, and mice assigned randomly to treatment arms. Treatment with **329** was initialized at day 6 and administered *via* intraperitoneal injection (IP) once daily (20 mg/kg), as indicated (**|**). Tumor volumes were completed with caliper measurement biweekly. **(B)** A549 cells were implanted subcutaneously, mice were randomized, and treatment with **329** was initiated at day 11 *via* IP (40 mg/kg) and treated once daily as indicated (|). **(C)** Tumor weight from A549 cells was determined on day 32. Statistically significance differences from vehicle-treated tumors are indicated by the asterisk *p < 0.05; ***p < 0.01.

The long-term goal is to move toward efficacious and safe combination therapies that include RPA inhibition. Predecessor molecules to **329** and **2004**, compounds 505 and 551, possessed modest *in vivo* activity (8). With optimized formulation for **329** and favorable PK parameters, we proceeded to *in vivo* single-agent efficacy studies in H460 large cell carcinoma and A549 adenocarcinoma xenografts. Tumor cells were implanted in NOD/SCID mice that were randomized and treated with vehicle or 20 mg/kg of **329**. Considering the rapid growth kinetics of H460, we administered **329** at 20 mg/kg daily for 5 days, with 2 days off, repeated 3 times. With this dosing strategy, a decrease in tumor volume was observed starting at day 17 (**Figure 8A**). Importantly, previous studies with **329** in a suboptimal formulation resulted in similar tumor growth delay, but with dosing completed at 200 mg/kg (data not shown). This suggests that the newly identified formulation results in single-agent activity as predicted, but that anticancer activity can be achieved using one-tenth the amount of drug. This study clearly shows that a dynamic range is possible and further demonstrates a dose response to **329** *in vivo*, particularly profound as tumor size slightly increases when animals had 2 days of recovery from drug dosing, followed by an immediate tumor reduction after dosing was resumed. Similar studies were conducted in A549 xenograft model, with IP dosing as indicated in the figure, at 40 mg/kg. The results demonstrate that mice in the treatment arm display a significant reduction in tumor growth (**Figure 8B**). This result was confirmed in the analysis of terminal tumor weight that revealed significant smaller tumors in **329**-treated mice (**Figure 8C**). Together, these data demonstrate the utility of RPAi in treating lung cancer. Further analysis including dosing and schedule is predicted to achieve maximal anticancer activity.

## Discussion

The DDR is actively being pursued for cancer therapy, with phase I results being recently reported for ATRis (15). The vast majority of individual targets being developed in the DDR space are kinases, largely a function of the advances made over the past decade on developing kinase-targeted agents in the growth signaling pathways (16). Kinases, however, represent a minority of the protein components in the DDR pathway and larger replication, repair, and recombination pathways [8;25]. There are myriad opportunities to impede the DDR *via* non-kinase targeted agents (10, 17, 18). The DDR pathway is initiated by sensing DNA discontinuities, damage, or DNA structures *via* DNA binding modules associated with each kinase DNA-PK, ATM, and ATR. We have targeted these unique protein–DNA interactions with small molecules to first elucidate specific mechanisms of DDR activation that can be used to guide the development of cancer therapeutics (19–23). RPA is a complex target as a function of its roles in multiple DNA metabolic and catabolic pathways (24). Two classes of RPAis were initially discovered: (i) covalent RPA modification agents and (ii) reversible inhibitors that target the oligonucleotide/oligosaccharide binding folds (OB-folds) responsible for the RPA–DNA interaction (3). In this report using optimized reversible RPAis, we demonstrate single-agent *in vivo* activity and synergy in combination with traditional and DDR-targeted therapy. Furthermore, we probed the putative mechanism of RPAi’s anticancer activity.

PARPi therapy has now been approved in 4 different solid tumors, with prostate and pancreatic joining ovarian and breast in the list of approved indications. Recent evidence on the mechanism of PARPi suggests that ssDNA and specifically lagging strand gaps contribute to PARP efficacy (25). If this mechanism is relevant, one could envision that BRCA wild-type cells would be sensitized to PARPi if the DDR was chemically inhibited. Our finding of synergy as measured by Chou-Talalay combination index analysis supports this basic finding and extends to our recent analyses in BRCA1-deficient cells that show that BRCA1-deficient cells are hypersensitive to RPAi compared to BRCA-complemented cells (26). The observation of synergy in BRCA wild-type cells suggests that RPA inhibition could impair homologous recombination repair (HRR) to create an HR phenotype that increases the potency of PARPi. The impact on HR could be in addition to the effect on the DDR. This result is consistent with our observation of synergy with both ATR and DNA-PK inhibition that can be explained by RPAi impacts on individual and parallel pathways or cross talk between the DDR signaling events. An alternative hypothesis is that another aspect of RPA involvement could explain the synergy, including an alteration in replication fork stability and restart. This is supported by the single-molecule studies that the effects of RPAi on replication dynamics are similar to those of ATRi effects that remain consistent with the dependent nature of ATR on RPA–DNA binding activity in signaling replication fork stress. It is interesting that ATR activity is impacted by ATM as well based on recent studies in both *in vitro* models and patient responses in clinical trial data. This suggests that the cross talk between the three arms of the DDR, DNA-PK, ATM, and ATR, is advantageous if not necessary for responding to replication stress or DNA damage. The ability to block the binding of RPA to ssDNA can induce differential effects depending on the RPA requirement for each pathway. For instance, the amount of RPA needed for nucleotide excision repair (NER) of cisplatin-treated cells is anticipated to be very low based on the cellular levels of cisplatin damage. Accordingly, our observation that RPAi does not impact NER-catalyzed repair is not surprising. Similarly, in normal, unperturbed DNA replication, RPAi has minimal effects on our initial assessment of replication dynamics; however, when fork stalling is induced by HU, a dramatic effect of RPAi is observed, consistent with the increase in the amount of RPA needed to address the replication stress and the limited RPA available as a function of the inhibitor.

The model of RPA threshold is consistent with our analysis of RPAi cellular activity and the tumor agnostic mechanism of action. Also consistent with these data are previous findings that RPA expression has been described as a prognostic and predictive biomarker in a small number of studies (27–29). Our retrospective analysis of NSCLC confirms and extends these studies to demonstrate that RPA expression levels can be both prognostic and predictive in smoking-associated lung cancers. Its role in the DDR is likely critical to respond and protect from the myriad of genetic insults stemming from carcinogen exposure. It is therefore interesting to speculate that RPA expression or activity may also be predictive of response to other DDR-targeted therapeutics.

Recent advances in kinase-targeted agents and immuno-oncology (IO) therapy have changed many treatment paradigms for lung cancer. The discovery of driver mutations and chromosomal rearrangements in NSCLC has resulted in the availability of molecularly targeted agents for 40% of NSCLC (30), including EGFR tyrosine kinase inhibitors (TKIs) and ALK TKIs. Despite these targeted therapeutic advances, the clinical reality is that over 60% of NSCLC patients will continue to receive the common chemotherapy, Pt-based agent, as part of their therapy. Lung epithelial cells are exposed to a variety of carcinogens that can be dramatically increased in cigarette smoke exposure and likely contribute to the high mutation burden observed in smoking-related cancers. It stands to reason that lung epithelium would have a robust DNA repair capacity to counter the DNA damage elicited by cigarette smoking, and early research demonstrated the importance of DNA repair in lung carcinogenesis (31, 32). This repair capacity can explain the rapid resistance to cancer therapeutic modalities that induce DNA damage including two frequently used Pt-based agents, cisplatin and carboplatin, and ionizing radiation. Recent advances in our understanding of how cells, both normal and cancerous, respond to DNA damage stress has identified a number of unique vulnerabilities that can be exploited for effective therapy to treat cancer. Our retrospective and cellular data strongly suggest that RPA plays an important role in treating this disease. This premise is supported by the clear single-agent anticancer activity observed with our newly formulated RPAi and combined suggests that inhibition of RPA will have a significant impact on cancer therapy in this difficult to treat disease.

## Data Availability Statement

The original contributions presented in the study are included in the article/supplementary material. Further inquiries can be directed to the corresponding authors.

## Ethics Statement

The animal study was reviewed and approved by IACUC-Indiana University.

## Author Contributions

PV-C contributed cellular studies. NG contributed chemical synthesis. EE, JH, and SP contributed DNA fiber analysis data. All authors contributed to the article and approved the submitted version.

## Funding

This work was supported by NIH grant R01 CA257430 and the Tom and Julie Wood Family Foundation (J.J.T.). Additional studies were supported by NIH grant RO1CA229535 (S.M.P.), NICHD P50HD090215 (K.E.P), P30CA082709 awarded to the Indiana University Simon Comprehensive Cancer Center (K.E.P), and the Indiana University Grand Challenge–Precision Health Initiative (K.E.P).

## Conflict of Interest

Author JT is a shareholder and founder and KP is a shareholder and employed by NERx BioSciences.

The remaining authors declare that the research was conducted in the absence of any commercial or financial relationships that could be construed as a potential conflict of interest.

## Publisher’s Note

All claims expressed in this article are solely those of the authors and do not necessarily represent those of their affiliated organizations, or those of the publisher, the editors and the reviewers. Any product that may be evaluated in this article, or claim that may be made by its manufacturer, is not guaranteed or endorsed by the publisher.
